# Phenotypic Characterization of 2D and 3D Prostate Cancer Cell Systems Using Electrical Impedance Spectroscopy

**DOI:** 10.3390/bios13121036

**Published:** 2023-12-18

**Authors:** Lexi L. Crowell, Juan Sebastian Yakisich, Brian Aufderheide, Tayloria N. G. Adams

**Affiliations:** 1 Department of Chemical and Biomolecular Engineering, University of California Irvine, Irvine, CA 92697, USA; crowelll@uci.edu; 2 Sue and Bill Gross Stem Cell Research Center, University of California Irvine, Irvine, CA 92697, USA; 3 Department of Pharmaceutical Sciences, School of Pharmacy, Hampton University, Hampton, VA 23668, USA; juan.yakisich@hamptonu.edu; 4 Department of Chemical Engineering, Hampton University, Hampton, VA 23668, USA; brian.aufderheide@hamptonu.edu; 5 Department of Biomedical Engineering, University of California Irvine, Irvine, CA 92697, USA

**Keywords:** microfluidics, epithelial-mesenchymal-transition, chemoresistance, cell plasticity, cell phenotype

## Abstract

Prostate cancer is the second leading cause of death in men. A challenge in treating prostate cancer is overcoming cell plasticity, which links cell phenotype changes and chemoresistance. In this work, a microfluidic device coupled with electrical impedance spectroscopy (EIS), an electrode-based cell characterization technique, was used to study the electrical characteristics of phenotype changes for (1) prostate cancer cell lines (PC3, DU145, and LNCaP cells), (2) cells grown in 2D monolayer and 3D suspension cell culture conditions, and (3) cells in the presence (or absence) of the anti-cancer drug nigericin. To validate observations of phenotypic change, we measured the gene expression of two epithelial markers, E-cadherin (CDH1) and Tight Junction Protein 1 (ZO-1). Our results showed that PC3, DU145, and LNCaP cells were discernible with EIS. Secondly, moderate phenotype changes based on differences in cell culture conditions were detected with EIS and supported by the gene expression of CDH1. Lastly, we showed that EIS can detect chemoresistant-related cell phenotypes with nigericin drug treatment. EIS is a promising label-free tool for detecting cell phenotype changes associated with chemoresistance. Further development will enable the detection and characterization of many other types of cancer cells.

## 1. Introduction

Prostate cancer is a life-altering disease, and the second leading cause of death in men [1]. Primary tumors in the prostate grow slowly and have a long latent period; however, once prostate cancer becomes chemoresistant, it is difficult to treat and contributes to high mortality rates [2]. Methods such as real-time quantitative polymerase chain reaction (RT-qPCR), Western blotting, flow cytometry, and next-generation sequencing are used to identify biomarkers related to the chemoresistance of prostate cancer cells [3,4,5]. The disadvantages to these methods include that they are difficult to perform, requiring trained technicians; they are time-consuming; expensive; and provide non-continuous monitoring of prostate cancer cell chemoresistance.

Chemoresistance is a critical challenge in the treatment of prostate cancer. Cancer cells have elusive properties that give rise to important dynamics; in particular, cancer cells can transition from being non-chemoresistant to chemoresistant, which is attributed to their plasticity. Cell plasticity is the ability of a cell to undergo reversible molecular and phenotypic changes in response to the microenvironment, and is linked to cancer cell chemoresistance [6,7,8]. For instance, chemical castration is effective in treating cancer cells for one to two years, and then castration-resistant cancer cells gradually develop. Several factors (i.e., phenotype changes) contribute to the plasticity of cancer cells, such as mutations in the androgen receptor and overexpression of the protein ABCG2, which in turn contributes to the production of chemical castration-resistant cancer cells [9]. Another phenotype change associated with chemoresistance is the epithelial mesenchymal transition (EMT) [10,11], a biochemical process in which cancer cells lose their epithelial features and gain mesenchymal features [12]. Sethi et al. found that E-cadherin, vimentin, PDGF-D, NF-kB, Notch-1, and ZEB1 (EMT markers) were present in primary prostate cancer tissue and bone metastasis [10]. In particular, Notch-1 was overexpressed in bone metastasis vs. primary prostate cancer tissue, suggesting that upregulation of Notch-1 plays a significant role in bone metastasis [10]. Similarly, Wang et al., found that the expression of Notch-1 played an important role in prostate cancer cells’ chemoresistance. Notable findings include that (a) PC3 and DU145 cell lines resistant to paclitaxel had downregulation of E-cadherin, upregulation of vimentin, and upregulation of Notch-1; (b) overexpression of E-cadherin in drug-resistant cell lines was associated with downregulation of vimentin and inhibited cell migration; and (c) the overexpression of E-cadherin in drug-resistant cell lines increased the effectiveness of paclitaxel [11]. Currently, 2- and 3-dimensional (2D monolayer, 3D suspension) cell culture models are used to understand the cellular dynamics involved in plasticity [13,14]. While correlating cell signaling pathways to phenotype changes is not in the scope of this work, it is important to highlight connections to chemoresistance.

Since plasticity is a major contributor to cancer cell chemoresistance, the detection and characterization of prostate cancer cell phenotypic changes is necessary. One detection method is electrical impedance spectroscopy (EIS), an electrode-based cell characterization technique that requires cell polarization, which induces cell movement (i.e., trapping between electrodes) [15,16,17]. While conventional cell characterization techniques (i.e., flow cytometry) rely on external labels to distinguish cell phenotypes, EIS is a label-free method that uses a set of intrinsic biomarkers (impedance and capacitance) to characterize cells. Compared to conventional label-based techniques, label-free methods minimize cell damage, are more cost effective, and require less labor-intensive processes [17].

EIS shows promise in understanding cancer cell dynamics and the efficacy of chemotherapeutics [15,16,17]. EIS theory has been reviewed [18]; briefly, during EIS measurements, an alternating current electric field is applied over a wide range of frequencies. When EIS is active, the electric field interacts with the cell membrane, membrane proteins, and intracellular molecules [19]. The resulting EIS response has unique frequency-dependent characteristics of transient and plateau regions. At radio frequencies (10^3^–10^8^ Hz), also known as the β-dispersion region, the transient region provides information about the surface charge mobility, and the plateau region provides information about cell membrane thickness [19]. The plateau region is also influenced by relaxation effects caused by proteins and amino acids residues, and internal organelles, primarily cell nuclei and mitochondria (termed Maxwell–Wagner effects) [19]. Therefore, for prostate cancer cells, selectively probing the β-dispersion region can reveal intrinsic and extrinsic factors that contribute to their phenotype changes linked to chemoresistance.

EIS has been used to discern many types of human and mouse cancer cells based on their electrical properties [15,20,21,22]. Teixeira et al. used EIS to characterize the impedance spectra of PC3 and DU145 cells [23]. They found that the impedance spectra for DU145 cells were higher than PC3 cells over a wide range of frequencies [23]. Drug-resistant breast cancer cells have been distinguished from parental cells using EIS [24]. Similarly, EIS was used to distinguish normal and cancerous lung cells [25], and this technology was used to monitor prostate cancer cells while they were subjected to changes in pH [26]. Additionally, EIS distinguishes lineage specification in other types of cancer cells [27], raising the possibility that cell biophysical measures are specific to chemoresistant cell subpopulations in many types of cancers.

Correlating biological assessments of prostate cancer cell phenotype changes to engineering assessments are important. We have categorized cell phenotype changes as large (differences in cell lines), moderate (differences in cell culture condition), and subtle (differences in cells day-to-day). Large phenotype changes were examined via three prostate cancer cell lines, PC3, DU145, and LNCaP. Moderate phenotype changes were mimicked by culturing the prostate cancer cells lines in a 2D monolayer and 3D suspension. Lastly, subtle phenotype changes were examined, with daily assessments of the cells.

In this study, the EIS of prostate cancer cells lines (PC3, DU145, and LNCaP) cultured as a 2D monolayer and 3D suspension were characterized. Our EIS results showed that the impedance spectra for DU145 cells were higher than the PC3 and LNCaP cells for the 2D monolayer and 3D suspension cell culture. We measured the impedance spectra at multiple time points (Day 1, 3, and 7), and were able to detect moderate changes in cell phenotype. On Day 3, the averaged impedance for DU145, PC3, and LNCaP cells changed, suggesting a phenotype change occurred. Epithelial phenotype markers, E-cadherin (CDH1) and Tight Junction Protein 1 (ZO-1), were quantified for the 2D monolayer and 3D suspension culture conditions. The relative expression of CDH1 supports the EIS detection of cell phenotype change. Subtle phenotype changes were difficult to confirm with relative gene expression. Finally, a nigericin drug test revealed that impedance measurements detect cell death, which can be potentially correlated with the chemoresistance of cancer cells based on viability. EIS provides label-free and noninvasive impedance measurements. These findings demonstrate the potential of EIS as a real-time monitoring tool for detecting phenotype changes in prostate cancer cells.

## 2. Materials and Methods

### 2.1. Cell Lines

Human prostate cancer cell lines, PC3, DU145, and LNCaP, were obtained from the American Type Culture Collection (ATCC, Manassas, VA, USA). Cells were grown in Roswell Park Memorial Institute 1640 (RPMI-1640) culture medium (ThermoFisher, Waltham, MA, USA) supplemented with 10% fetal bovine serum (FBS, Corning, Corning, NY, USA) and 1% penicillin–streptomycin (Fisher Scientific, Hanover Park, IL, USA).

For 2D monolayer cell culture, the cells were grown in a 5% CO_2_ incubator at 37 °C and passaged at ~80% confluence. To passage, the cells were washed with 1X DPBS (Life Technologies, Carlsbad, CA, USA) and incubated with 0.05% trypsin-EDTA (Life Technologies, Carlsbad, CA, USA) for 7 min at room temperature. RPMI-1640 was added to neutralize the trypsin and prevent over-digestion. The detached cell suspension was centrifuged for 5 min at 130× *g* and resuspended in a new growth medium.

For 3D suspension cell culture, the cells were plated in a T75 flask with RPMI-1640. The cells were grown as a monolayer until ~80% confluent, trypsinized, and plated with fresh RPMI-1640 in a 6-well ultra-low attachment (ULA) plates (Corning, Corning, NY, USA). The cells were closely observed for 24 h to ensure spheroid formation. The RPMI-1640 was replaced twice a week by collecting suspended cells in 15 mL Falcon tubes, centrifuging (130× *g* for 5 min) and discarding the supernatant. The cells were resuspended in 10 mL of fresh RPMI-1640 and transferred to their original 6-well ULA plates. This protocol was adapted from [28].

### 2.2. Nigericin Treatment

To further assess phenotype changes in the 2D monolayer and 3D suspension cultures, the PC3 cells were treated with nigericin (Fisher Scientific, Hanover Park, IL, USA). A stock solution of nigericin was prepared in dimethyl sulfoxide (DMSO, 2.5 mM), aliquoted, and stored at −20 °C. The 2D monolayer and 3D suspension cultures were initiated and maintained until the cells were ~80–90% confluent. Then, 24 h later, separate wells of PC3 cells were treated with 0 μM, 5 μM, and 10 μM nigericin. The 0 μM nigericin solution only contained DMSO (10 μL). The cells were incubated with nigericin at 5% CO_2_ and 37 °C for 48 h and 96 h, and the viability was checked using trypan blue exclusion dye. After the nigericin treatment, the cells were prepared for EIS and gene expression analysis.

### 2.3. EIS Characterization

On the day of the experiment, cells were trypsinized and resuspended in a low-conductivity buffer (LCB) solution consisting of 8.5% (*w*/*v*) sucrose and 0.3% (*w*/*v*) glucose adjusted to a final conductivity of 100 μS/cm by adding RPMI-1640 medium. The cells were washed 3 times in the LCB, and approximately 300,000 cells were prepared for EIS characterization. To perform EIS, a microwell device with parallel electrodes (fabricated using previously published techniques [29,30]; electrode dimensions: 50 μm wide with 100 μm spacing) was washed 3 times with the LCB and connected to a Reference 600+ Potentiostat/Galvanostat/ZRA (Gamry Instruments, Warminster, PA, USA). A full EIS characterization was performed using a frequency sweep from 100 Hz to 10 MHz at 10 mV for 5 min, and 3 runs were completed for each independent experiment. An averaged impedance was calculated by taking the mean of the impedance values from 10^2^ Hz to 10^6^ Hz. As a blank, the LCB was run 3 times with the Reference 600+ Potentiostat/Galvanostat/ZRA.

### 2.4. Gene Expression Analysis

**Figure 1:**
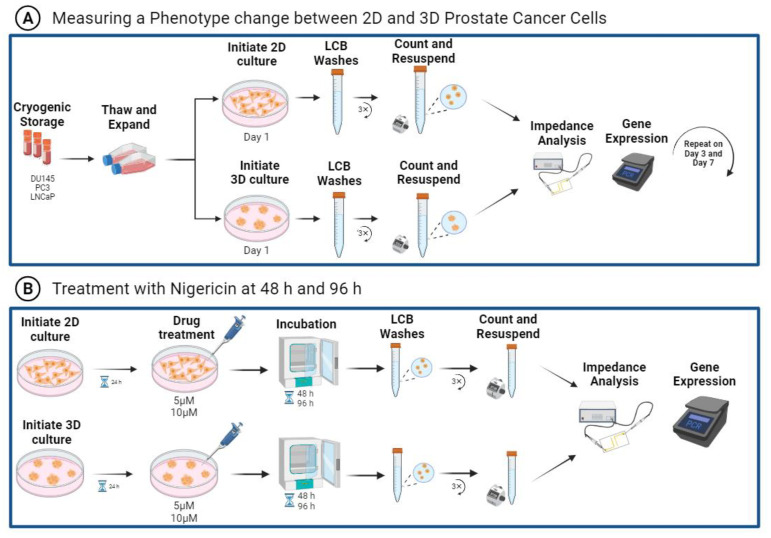
EIS experimental workflow for characterizing phenotype changes in prostate cancer cells. (**A**) DU145, PC3 and LNCaP cells were obtained from cryogenic storage, thawed, and expanded in proliferation media. About 300 K cells of 2D monolayer and 3D suspension growth were prepared in LCB solution for impedance analysis, while the remaining cells were used for gene expression. (**B**) Nigericin was added to PC3 cells cultured as 2D monolayer and 3D suspension for 24 h after seeding. The cells were measured for gene expression and impedance 48 h and 96 h after treatment. Figure created with BioRender.com.

RT-qPCR was used to quantify changes in gene expression for 2D monolayer and 3D suspension cell cultures of PC3, DU145, and LNCaP cells. RT-qPCR was also used to identify changes in gene expression of PC3 cells with the treatment of nigericin. RNA was extracted using an RNA micro prep kit (Qiagen, Germantown, MD, USA). The growth medium was aspirated, and RNA was extracted from the samples by adding the lysis buffer and purified following the manufacturer’s protocol. cDNA was synthesized using the Lunascript reverse transcription master mix (New England Biolabs, Ipswich, MA, USA). CDH1 and ZO-1 genes were assessed to characterize phenotype changes. Relative quantification of mRNA expression was obtained using the ΔΔCt method, with the target genes normalized to GAPDH as the endogenous control. Figure 1 outlines the experimental workflows described. Primer sequences can be found in Supplemental Table S1.

### 2.5. Statistical Analysis

**Figure 2:**
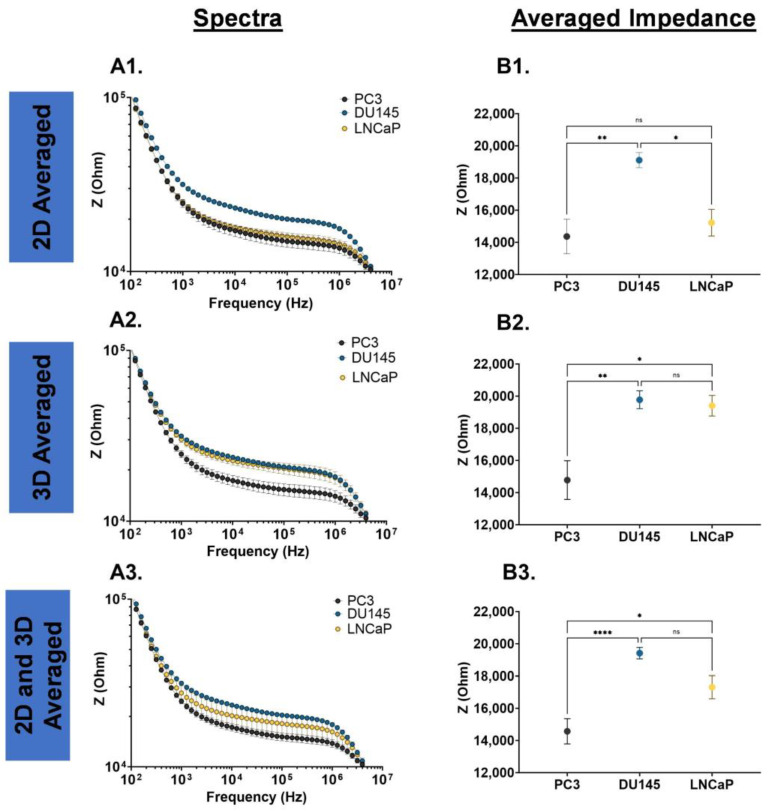
EIS cell analysis for PC3, DU145, and LNCaP cells. (**A1**) Cells grown as a 2D monolayer, (**B1**) cells grown as a 3D suspension, and (**C1**) average values of the 2D monolayer- and 3D suspension-cultured cells. (**A2**,**B2**,**C2**) Averaged impedance values from 10^3^ Hz to 10^6^ Hz. Statistical analysis completed on pooled data sets. *n* = 3; * *p* < 0.05, ** *p* < 0.01 and **** *p* < 0.0001.

**Figure 3:**
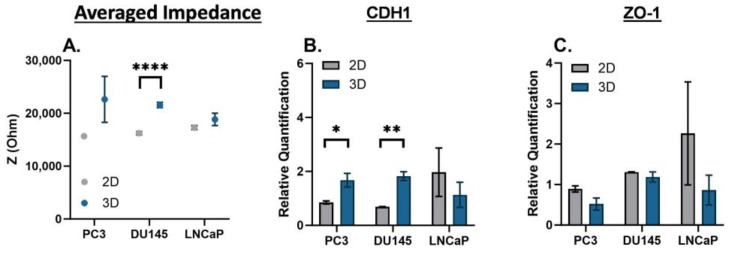
EIS and gene expression of PC3, DU145, and LNCaP cells cultured as a 2D monolayer and 3D suspension at Day 3. (**A**) Averaged impedance values from 10^3^ Hz to 10^6^ Hz. Gene expression of (**B**) CDH1 and (**C**) ZO-1. Statistical analysis completed on pooled data sets. *n* = 3; * *p* < 0.05, ** *p* < 0.01 and **** *p* < 0.0001.

**Figure 4:**
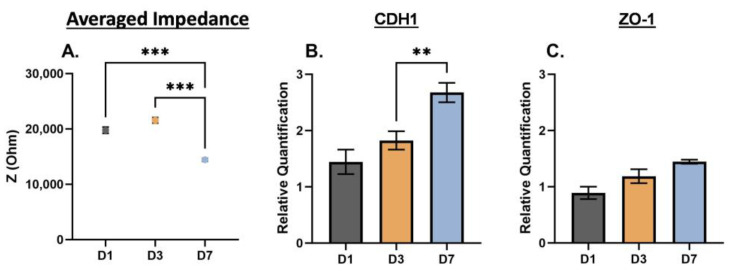
EIS and gene expression of DU145 cells cultured in 3D suspension at Day 3. (**A**) Averaged impedance values from 10^3^ Hz to 10^6^ Hz. Gene expression of (**B**) CDH1 and (**C**) ZO-1. D1 = Day 1, D3 = Day 3, and D7 = Day 7. Statistical analysis completed on pooled data sets. *n* = 3; ** *p* < 0.01; *** *p* < 0.001.

**Figure 5:**
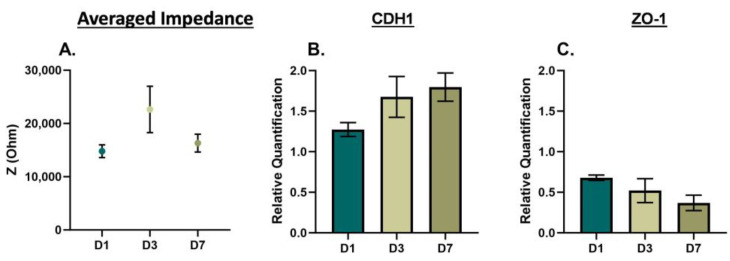
EIS and gene expression of PC3 cells cultured in 3D suspension on Day 3. (**A**) Averaged impedance values from 10^3^ Hz to 10^6^ Hz. Gene expression of (**B**) CDH1 and (**C**) ZO-1. D1 = Day 1, D3 = Day 3, and D7 = Day 7. Statistical analysis completed on pooled data sets. *n* = 3.

Statistical analysis for the EIS characterizations of 2D monolayer and 3D suspension cultures of PC3, DU145, and LNCaP cells were completed using an ordinary one-way ANOVA with Tukey’s multiple comparisons test (Figure 2, Figure 4 and Figure 5, and Supplemental Figure S4). The EIS and gene expression characterization of 2D monolayer and 3D suspension cultures of PC3, DU145, and LNCaP cells at Day 3 were assessed using unpaired *t*-tests (Figure 3). The statistical analysis of the nigericin treatment data was completed using a two-way ANOVA with Dunnett’s multiple comparisons test. Biological replicates are listed as “*n*” in the figure legends. For statistically significant data, * signifies *p* < 0.05, ** signifies *p* < 0.01, *** signifies *p* < 0.001, and **** signifies *p* < 0.0001. Error bars represent the standard error mean; some are too small for the bars to be visible.

## 3. Results

EIS characterization is a rapid method enabling the assessment of various cell types under different culture conditions in a high-throughput manner, all using the same operating parameters. EIS cell analysis of prostate cancer cells was completed using frequency sweeps from 100 Hz to 10 MHz, and Figure 1 outlines the experimental workflow. Supplemental Figure S1 illustrates the dielectric dispersions experienced by cells with EIS measurements. We characterized phenotype changes in prostate cancer cells by examining PC3, DU145, and LNCaP cells in 2D monolayer cultures and 3D suspension cultures, and comparing their CDH1 and ZO-1 gene expression, Figure 1A. The EIS measurements were repeated over seven days to examine phenotype change with time, as shown in Figure 1A. To further illustrate EISs’ potential to detect phenotype changes in prostate cancer cells, nigericin treatments were performed. The averaged impedance spectra, CDH1, and ZO-1 gene expression were assessed, as shown in Figure 1B. Additionally, Supplemental Figure S2 includes a schematic of the microwell device, with parallel electrodes used for EIS measurements and an illustration of the Bode and Nyquist plots generated.

Prostate cancer cells may experience phenotype changes based on internal (genetic and epigenetic changes) and external (microenvironment changes) factors; thus, we examined the impedance spectra of PC3, DU145, and LNCaP cells cultured as a 2D monolayer and 3D suspension. The impedance spectra of these cells were defined by two transitions (10^2^–10^3^ Hz and 10^6^–10^7^ Hz) and a plateau (10^3^–10^6^ Hz). In Figure 2(A1,A2), the DU145 cells have a higher impedance magnitude over the entire frequency range tested, and a higher averaged impedance, which was statistically significant. In the 3D suspension cell culture system, as shown in Figure 2(B1,B2), the DU145 and LNCaP cells have a higher magnitude and averaged impedance in comparison to PC3 cells, which were statistically significant. The impedance spectra for the PC3, DU145 and LNCaP cells grown as a 2D monolayer and 3D suspension were averaged over the entire frequency range tested (Figure 2(C1)), and over the plateau (Figure 2(C2)). In both cases, the DU145 has the highest impedance magnitude and averaged impedance, followed by LNCaP and PC3 cells (DU145 > LNCaP > PC3).

There is a time dependency on the phenotype changes that prostate cancer cells may experience. Therefore, we compared the 2D monolayer and 3D suspension cultures on Day 3. The averaged impedance and gene expression of CDH1 and ZO-1 are shown for PC3, DU145, and LNCaP cells in Figure 3. For each prostate cancer cell line, the averaged impedance was higher for 3D suspension cultures versus 2D monolayer cultures, and statistically significant for the DU145 cells (**** *p* < 0.0001); the full impedance spectra are included in Supplemental Figure S3. The relative gene expression of CDH1 was higher for the 3D suspension cultures of PC3 and DU145 cells (* *p* < 0.05 and ** *p* < 0.01, respectively) and lower for the LNCaP cells. The relative gene expression of ZO-1 was lower for the 3D suspension cultures for all prostate cancer cell lines.

To further examine phenotype changes in prostate cancer cells based on culturing conditions, we repeated the EIS cell analysis over seven days, with time points at Day 1 (D1), Day 3 (D3), and Day 7 (D7) for 3D cells in suspension. The averaged impedance and gene expression of CDH1 and ZO-1 are shown for DU145 cells in Figure 4, for PC3 cells in Figure 5, and for LNCaP cells in Figure S4. For the DU145 cells, there was an increase and decrease in the averaged impedance, as shown in Figure 4A, yielding an overall decrease in impedance (D1 to D7 and D3 to D7, *** *p* < 0.001). The relative gene expression of CDH1 and ZO-1, as shown in Figure 4B,C increased from D1 to D7. For PC3 cells, there was an increase and decrease in the averaged impedance, as shown in Figure 5A, yielding an overall decrease in impedance. The relative gene expression of CDH1 increased and ZO-1 decreased from D1 to D7, as shown in Figure 5B,C. For the LNCaP cells, there was an overall decrease in impedance (D1 to D7, * *p* < 0.05), and the relative gene expression of CDH1 stayed consistent, while ZO-1 decreased. The full EIS spectra for DU145, PC3, and LNCaP cells over the seven-day culture period can be found in Supplemental Figure S5.

**Figure 6:**
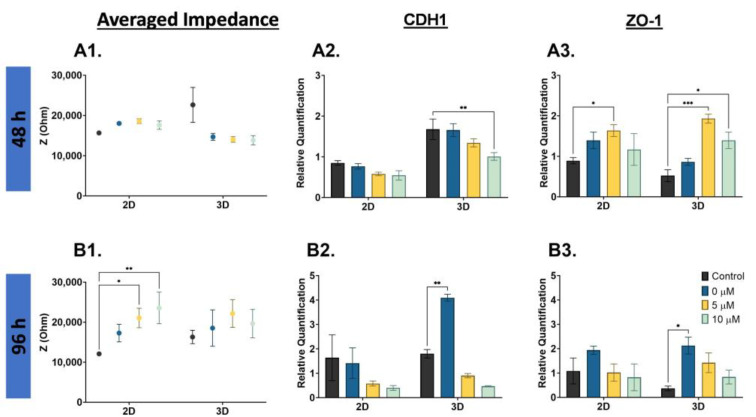
EIS and gene expression of PC3 cells treated with nigericin at 48 h (**top**) and 96 h (**bottom**) for the 2D monolayer and 3D suspension culture conditions. (**A1**,**B1**) Averaged impedance values with drug treatment, (**A2**,**B2**) CDH1, (**A3**,**B3**) ZO-1. Drug treatment concentrations reported for control: 0 µM, 5 µM, 10 µM. The 48 h control was measured on D3. The 96 h control was measured on D7. The 0 µM concentration included only DMSO. Statistical analysis completed on pooled data sets. *n* = 3; * < 0.05, ** *p* < 0.01, *** *p* < 0.001.

There is evidence that D3 is a critical time point for cancer cells’ phenotype change [28], and impedance increased at D3 for the PC3 cells; thus, we completed a nigericin drug treatment (0, 5, 10 µM) and EIS cell analysis on PC3 cells. The averaged impedance and CDH1 and ZO-1 gene expression are shown for 48 h and 96 h drug-treated PC3 cells in Figure 6. The averaged impedance shown compares the 2D monolayer and 3D suspension cultures for each concentration at 48 h (Figure 6(A1)), and there were no significant changes in impedance. At 96 h, the impedance for both the 2D monolayer and 3D suspension cells increased with increased nigericin concentration, as shown in Figure 6(B1). The full impedance spectra are compared in Figure S6. With the 96 h nigericin treatment, the control compared to 5 µM was statistically significant (* *p* < 0.05), and the control compared to 10 µM was statistically significant (** *p* < 0.01). For the 2D monolayer and 3D suspension cells, the CDH1 gene expression decreased with the nigericin drug treatment, Figure 6(A2). The lowest expression level was 10 µM, and was statistically significant when compared to the D7 control (** *p* < 0.01). A similar trend was observed at 96 h for CDH1 gene expression; the impedance increased with increased nigericin concentration. The control compared to 0 µM (only DMSO) was statistically significant (** *p* < 0.01), as shown in Figure 6(B2). The gene expression of ZO-1 increased with higher nigericin drug concentrations and decreased at 10 µM for the 2D monolayer, and decreased with higher nigericin drug concentrations for the 3D suspension cells. The 2D control compared to 5 µM was statistically significant (* *p* < 0.05), the 3D control compared to the 5 µM was statistically significant (*** *p* < 0.001), and the 3D control compared to the 10 µM was statistically significant (* *p* < 0.05) at 48 h, as shown in Figure 6(A3). There was not a noticeable trend in the 96 h ZO-1 gene expression in the 2D monolayer cells; however, in the 3D suspension cells, there was a decrease in ZO-1 as the concentration increased, as shown in Figure 6(B3). The 3D control compared to the 0 µM was statistically significant (* *p* < 0.05).

## 4. Discussion

Characterizing the electrical signature of phenotype changes that prostate cancer cells may experience is important because it can be linked to chemoresistance. EIS is a good cell analysis technique because it utilizes electric fields and cell polarization to detect phenotype changes in cells. EIS can discern cell phenotype changes resulting from internal and external factors by measuring impedance. The selection of PC3, DU145, and LNCaP prostate cancer cell lines stem from their well-documented differences in metastatic potential [31,32,33] and varying quantities of cancer stem-like cells [34]. Additionally, these cells are commonly used in prostate cancer cell research [35]. Thus, the scope and findings of this study were focused on large (metastatic, Figure 2), moderate (2D monolayer vs. 3D suspension cell cultures, Figure 3), and subtle (day-to-day, Figure 4 and Figure 5) phenotype changes in prostate cancer cells, and correlations to chemoresistance (Figure 6). The novelty of this study is based on label-free engineering measurements of impedance combined with gene expression for the detection of prostate cancer cell phenotype changes related to chemoresistance.

### 4.1. The Biophysical Meaning of Averaging the Impedance Spectra

In processing our impedance data, we averaged the plateau portion of the impedance spectra, which, from EIS theory, provides information about the cell membrane thickness [19]. As such, it is possible that several biochemical processes related to cell membrane dynamics (the EMT phenotype change) and the chemoresistance of cancer cells were averaged. These biochemical processes include loss of epithelial cell–cell junctions, changes in cell shape driven by reorganization of the cytoskeleton, crosstalk between signaling pathways, translational and post-translational regulation, and increased cell protrusions. The loss of tight junctions during EMT is associated with a decrease in the expression of tight junction protein ZO-1 due to diffusion of claudin and occludin from cells in contact. There is also a decrease in CDH1 due to destabilization of adherent junctions. During the destabilization process, CDH1 is cleaved at the cell plasma membrane, and subsequently degraded [36]. These biochemical processes will be unique or different prostate cancer cell lines (as suggested by our data), and are worth noting in the context of our data analysis.

### 4.2. EIS Distinguishes Large (Metastatic) and Moderate (2D Monolayer vs. 3D Suspension) Phenotype Changes

Our initial characterization of prostate cancer cell lines revealed that the impedance spectra and averaged impedance of DU145 cells were higher than PC3 and LNCaP cells. This trend was true for DU145 cells cultured as a 2D monolayer, 3D suspension, and the average of both culture conditions (Figure 2), and is in good agreement with the literature [23,26]. DU145 and PC3 cells correspond to advanced stages of prostate cancer (stage 1 and stage 4, respectively) [32,33], whereas LNCaP cells represent an earlier cancer [31]; additionally, these cell lines exhibit varying proportions of cancer stem-like cells [34]. Early and advanced-stage prostate cancer cells are resistant to chemotherapy drugs, indicating the presence of chemoresistant cells. Thus, our impedance measurements correspond well with the biology of DU145, PC3, and LNCaP cells. Similar impedance trends (i.e., fewer metastatic cells have higher impedance) have been found for breast [37], lung (inferred from capacitance) [38], and throat (inferred from admittance) [39] cancer cells.

Prostate cancer cells may experience moderate phenotype changes based on internal (genetic and epigenetic changes) and external (microenvironment changes) factors; thus, we examined the impedance spectra of PC3, DU145, and LNCaP cells cultured as a 2D monolayer and 3D suspension as a moderate phenotype change. Cells grown as a monolayer versus suspension mimic a phenotype change, where suspension cells are known to be more chemoresistant than monolayer cells [14,34]. In our results, the impedance spectra maintained their s-shapes, as shown in Supplemental Figure S3, and the difference in cell culture conditions was discernible, with some similar trends in the averaged impedance and gene expression.

### 4.3. EIS Distinguishes Subtle (Day-to-Day) Phenotype Changes in Prostate Cancer Cells

When studying phenotype change, it is important to observe change over a period of time. We studied PC3, DU145, and LNCaP cells in 3D suspension cultures for up to seven days. We were particularly interested in changes that occurred after three days of culturing because Kaushik et al. found that lung (H460), prostate (LNCaP), and breast (MCF-7) cancer cells grown in anchorage-independent cell culture conditions (i.e., suspension cells) developed chemoresistance to hydroxyurea, colchicine, and obatoclax in within three days, signifying a phenotype change [28]. For the DU145 cells, there may have been a slight phenotype change over the seven days, indicated by a decrease in averaged impedance at D7 and an increase in CDH1 at D7. We observed differences in the averaged impedance and the relative gene expression of CDH1 and ZO-1 for the PC3 cells. However, PC3 cells are mesenchymal in morphology, and represent stage 4 prostate cancer [33], making them less susceptible to subtle phenotype changes. Lastly, for the LNCaP cells, there may have been a slight phenotype change within the cells over the seven days, indicated by overall decrease in impedance; however, this was more difficult to confirm with gene expression (CDH1 stayed the same, and ZO-1 decreased).

Overall, impedance measurements can be used to identify changes in cells over time, which can be validated with gene expression. While the gene expression of CDH1 and ZO-1 may not be the most representative of a phenotype change, they are good indicators of epithelial (or lack of epithelial) morphology. All the impedance measurements were completed in the β-dispersion region and compared to CDH1 and ZO-1 gene expression. At low frequencies, the cell membrane acts as an insulating barrier (i.e., resistive pathway), and at high frequencies, current passes through the cell (i.e., capacitive pathway). We report averaged impedance values over the plateau region of the spectra coupling the resistive and capacitive properties of the cells for phenotype change detection. Our measurements were completed in a label-free manner, and we were able to see differences in cancer cell phenotype via the resistive and capacitive properties of the cancer cells. This is important in monitoring cancer cell dynamics and chemoresistance.

### 4.4. Chemoresistance of PC3 Cells

Detection of phenotype changes in cancer cells corresponding to chemoresistance is the goal of this study. Hence, the PC3 cells cultured as a 2D monolayer and 3D suspension were treated with nigericin, an ionophore that impacts the calcium channels on the surface of cancer cells [40,41]. Due to literature evidence suggesting that D3 is a critical moment for phenotype change with implications on chemoresistance, the impedance and viability measurements were completed on D3. The 48 h nigericin treatment did not have a significant impact on the impedance and gene expression of PC3 cells. The 3D suspension cells have slightly lower impedance and overall higher CDH1 gene expression, providing evidence that at D3, these cells may be more chemoresistant than the 2D monolayer cells. The higher expression of CDH1 corresponds to results from Sethi et al. [10] and Wang et al. [11]; CDH1 was upregulated in more chemoresistant cell lines. However, the 96 h nigericin treatment decreased the viability of the PC3 cells in the 2D monolayer and 3D suspension, associated with an increased impedance and decreased CDH1 and ZO-1 gene expression. These results reveal that the PC3 cells have a resistance to drug treatment which may be attributed to the mesenchymal state of the cells (i.e., these cells have already gone through a phenotype change such as the EMT).

### 4.5. Limitations and Clinical Relevance of Impedance Measurements

While our study has provided valuable insights into the use of EIS for the detection of phenotype changes in cancer cells, it is important to note the limitations. In this study, we primarily focused on three prostate cancer cell lines that demonstrate the feasibility of microfluidic impedance measurements in detecting large and moderate phenotype changes. This work was limited by the detection of subtle phenotype changes. These limitations may be addressed by modifying the surface of our sensing electrodes with gold nanoparticles combined with aptamers [42,43] to increase sensitivity and specificity enough to detect subtle day-to-day changes in cancer cells. The translation of our findings into a clinical setting may require additional improvements on EIS technology to ensure adequate sample preparation and developing a point-of-care device.

Despite these limitations, our findings are relevant to the clinic in several ways. Studying chemoresistant phenotypes with EIS can shed light on when cancers undergo EMT and the mechanism and time frame in which this phenomenon takes place. Detecting phenotype changes is significant in drug screening studies wherein changes in cell phenotype reflect how well (or not well) a drug is performing. More specifically, drug development focused on targeting cancer cells prone to undergoing EMT may be of interest to pharmaceutical industries.

Furthermore, EIS has broader clinical applications, including assessing the metastatic potential of cancer cells through cell detachment. Here, we mimicked phenotype changes linked to cell detachment with EIS of the 2D monolayer compared to 3D suspension cell culture systems. Another vital clinical application of EIS is its extension to liquid biopsies. EIS coupled with other electrokinetic techniques such as dielectrophoresis allows for fresh patient clinical samples to be processed (cancer cells separated from noncancerous cells) and monitored for circulating tumor cells before and after drug treatments. EIS and dielectrophoresis facilitate the bioprocessing of cancer cells in a label-free manner. Thus, using the electrical signature of cancer cells may provide more insight to phenotypic characterization.

## 5. Conclusions

EIS is a good, label-free method that can be used to identify the cytological stages of prostate cancer, detect a phenotype change, and measure drug resistance. This was observed in our experimental data using three prostate cancer cell lines: DU145, PC3, and LNCaP cells. While using 2D monolayer cell culture is important, 3D suspension cell culture is better for understanding cancer cell dynamics, as it better reflects physiological tissues. The simplicity of the impedance device (planar parallel electrodes and the PDMS microwell) allows for both 2D monolayer and 3D suspension cell characterization as well as real time monitoring of cells. Moreover, 3D suspension cells are more advantageous to use because they mimic the tumor microenvironment, especially when modeling chemoresistance and drug treatments. Variations in EMT-associated gene expression profiles depend on cell and tissue type and on the degree of progression towards a mesenchymal phenotype.

Future investigations will include increasing the sensitivity of impedance sensing electrodes and including more PCR markers to further identify if 3D suspension cells have undergone a phenotype change. Furthermore, considering the connection between chemoresistance and EMT, we will modulate the EMT, employing PCR markers and electron microscopy imaging to validate the potential of impedance in detecting phenotype changes associated with EMT. EIS allows for real time monitoring, which can also reveal when in time chemoresistance occurs. The proteins on the surface, phenotype change, and the timing factor for chemoresistance are all significant factors in cancer research, and can help to better understand cancer cell dynamics. These impedance investigations and modulation of the EMT will be completed on newer patient samples representing multiple types of cancer. The objective is to gain deeper insights into cancer cell dynamics concerning phenotype changes and chemoresistance, further validating EIS as a reliable tool for monitoring phenotypic alterations. EIS allows for the measurement of dynamic changes in the cell membrane and cytoplasm, and our results show promise in understanding these cell dynamics and the efficacy of chemotherapeutics.

## Data Availability

Data are contained within the article and Supplementary Material.

## Supplementary Materials

The following supporting information can be downloaded at: https://www.mdpi.com/article/10.3390/bios13121036/s1, Table S1: Primer information; Figure S1: (A1) The single-shell spherical model for cells, (B1) relative permittivity and specific conductivity based on the frequency ranges in the α, β, and γ region; Figure S2: Schematic of (A) microwell device with parallel electrodes used for EIS. The parallel electrodes were 50 μm wide and spaced 100 μm apart. (A1) Top view of microwell device without cells, connected to a circuit which yields a lower impedance measurement indicated with the associated (A2) Nyquist and (A3) Bode plots. (A4) Top view of microwell device with cells, connected to a circuit which yields a higher impedance measurement, indicated with the associated (A5) Nyquist and (A6) Bode plots; Figure S3: EIS of (A1) PC3, (A2) DU145, and (A3) LNCaP cells cultured as 2D monolayer and 3D suspension cells; Figure S4: EIS and gene expression of LNCaP cells cultured as 3D suspension at multiple time points. (A1) Averaged impedance values from 10^2^ Hz to 10^6^ Hz. Gene expression of (A2) CDH1 and (A3) ZO-1; Figure S5: EIS spectra of (A1) PC3, (A2) DU145, and (A3) LNCaP cells cultured in 3D conditions overtime; Figure S6: EIS spectra of PC3 cells treated with 0 μM, 5 μM, 10 μM of nigericin. (A1) 2D monolayer cells after the 48 h drug treatment. (A2) 2D monolayer cells after the 96 h drug treatment. (B1) 3D suspension cells after the 48 h treatment. (B2) 3D suspension cells after the 96 h treatment.
