# Supplementary material for: Phenotypic Characterization of 2D and 3D Prostate Cancer Cell Systems Using Electrical Impedance Spectroscopy
Source: Biosensors (Basel). 2023 Dec 18;13(12):1036. doi: 10.3390/bios13121036 (PMC10742279; doi:10.3390/bios13121036)
Supplement: Supplementary file 1 [file biosensors-13-01036-s001.zip › biosensors-2736882-supplementary.pdf]

Supplemental Information

The primer sequences are provided in Supplemental Table S1.

Table S1. Primer information

Target	FWD Primer 5' - 3'	REV Primer 5' - 3'	IDT Assay ID
GAPDH	GACAGTCAGCCGCATC TTCT	GCGCCCAATACGACCA AATC	-
CDH1	-	-	Hs.PT.58.3324071
CDH2	-	-	Hs.PT.58.26024443
TJP1	-	-	Hs.PT.58.2456962

The Forward and reverse primer pair information for qPCR gene expression assay. In the case where commercially available primer pairs were used, the assay ID is supplied in place of the actual primer sequences.

The dielectric dispersion regions with the corresponding polarization mechanism. This study probes the β-dispersion region.

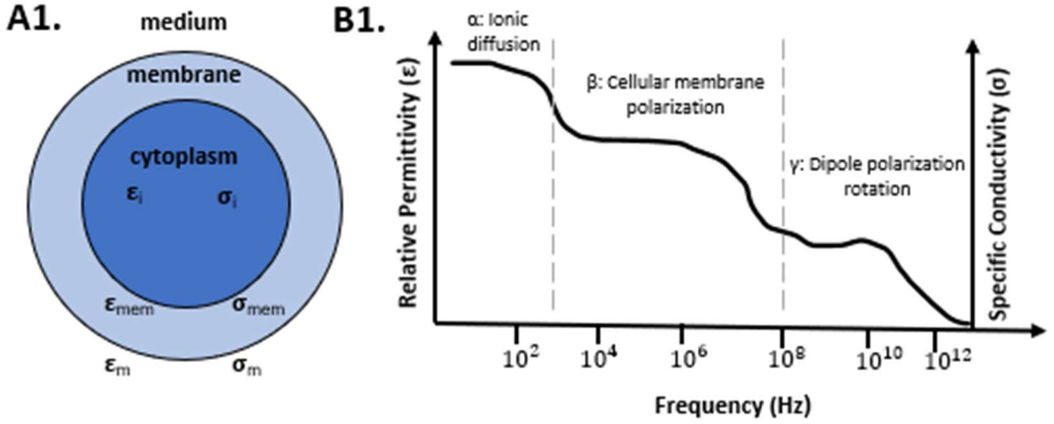

Figure S1. (A1) The single shell spherical model for cells, (B1) relative permittivity and specific conductivity based on the frequency ranges in the α, β, and γ region.

The design of the microwell device used in this study is provided. EIS is frequency dependent, resulting in imaginary and real values of resistance. Our EIS characterizations were performed using a frequency sweep, 100 Hz to 10 MHz for 5 minutes, at 10 mV. These measurements yield Nyquist and Bode plots which we have used to characterize phenotypic changes in prostate cancer cells.

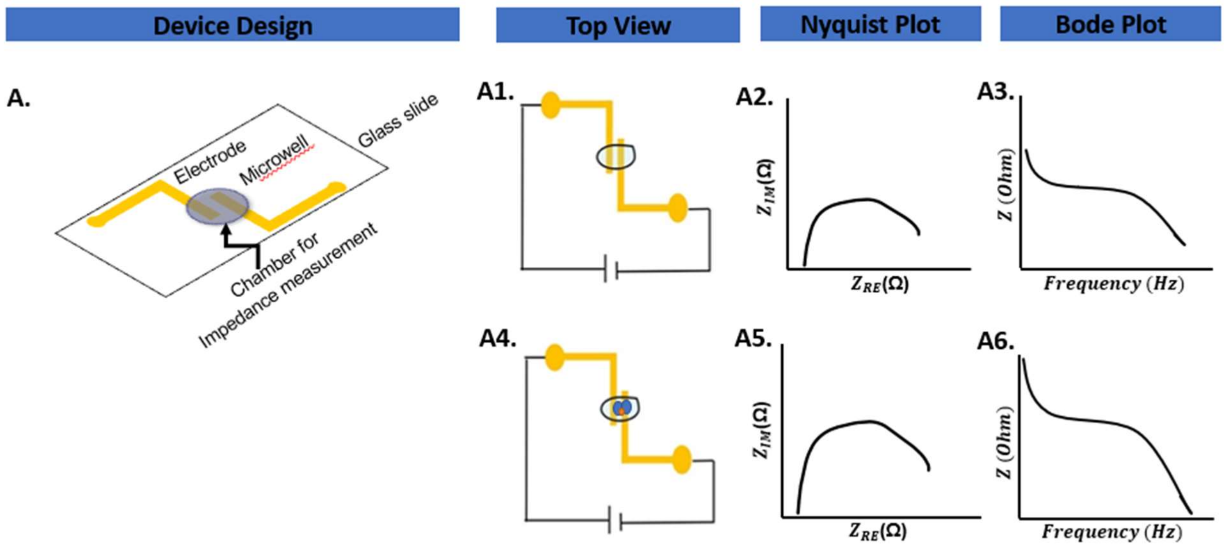

Figure S2. Schematic of (A) microwell device with parallel electrodes used for EIS. The parallel electrodes were 50 μm wide and spaced 100 μm apart. (A1) Top view of microwell device without cells, connected to a circuit which yields a lower impedance measurement indicated with the associated (A2) Nyquist and (A3) Bode plots. (A4) Top view of microwell device with cells, connected to a circuit which yields a higher impedance measurement indicated with the associated (A5) Nyquist and (A6) Bode plots.

Moderate phenotype changes, 2D monolayer and 3D suspension cultures of PC3, DU145, and LNCaP cells on Day 3, were characterized in Fig. 3. The EIS was reported as an average impedance. Here the average impedance spectra are provided. The PC3 and DU145 cell have the largest difference in impedance spectra for the moderate phenotype change. The impedance spectra for LNCaP cells were similar.

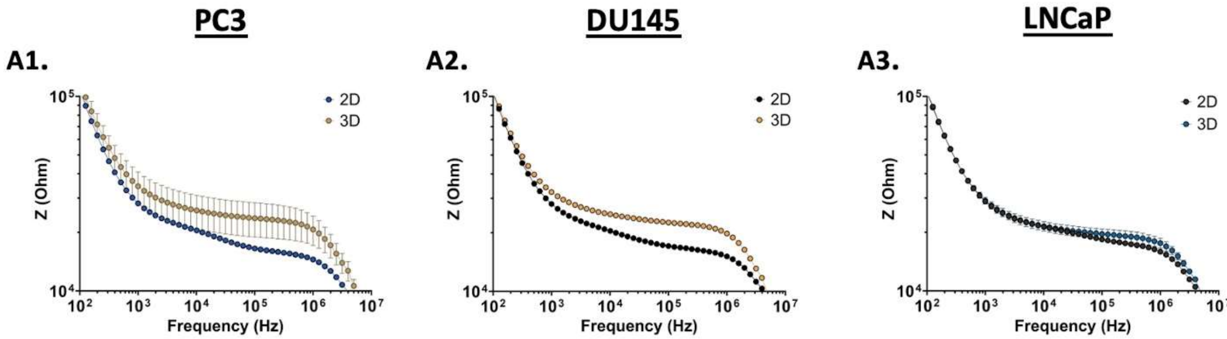

Figure S3. EIS of (A1) PC3, (A2) DU145, (A3) LNCaP cells cultured as 2D monolayer and 3D suspension cells. The EIS measurement occurred on Day 3. For PC3 and DU145 cells, n = 3 and for the LNCaP cells, n=2.

Subtle phenotype changes, day-to-day differences, of LNCaP cells were characterized. The cells were cultured as 3D suspension for seven days and the average impedance and relative gene expression were quantified. There was an overall decrease in impedance and ZO-1 while the gene expression of CDH1 stayed relatively consistent.

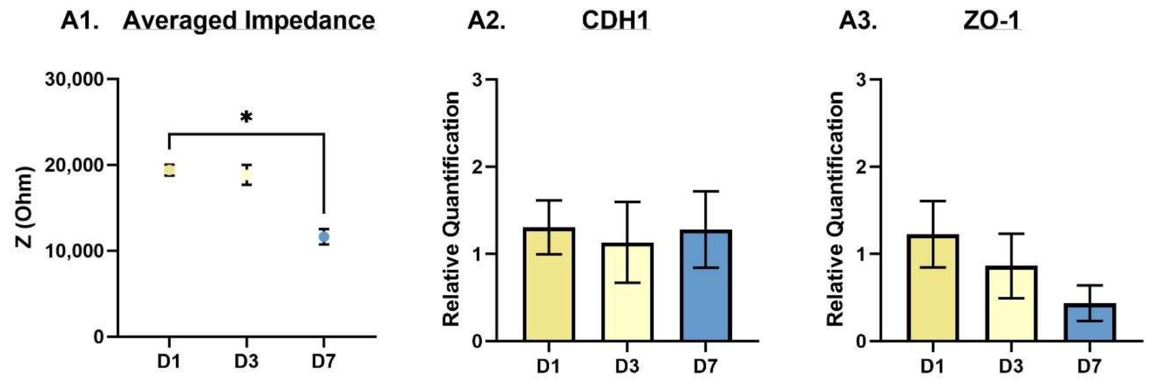

Figure S4. EIS and gene expression of LNCaP cells cultured as 3D suspension measured at multiple time points. (A1) Averaged impedance values from 10^2 Hz to 10^6 Hz. Gene expression of (A2) CDH1 and (A3) ZO-1. Statistical analysis was completed on pooled data sets, n = 3; *<0.05. Abbreviations: D1 = Day 1, D3 = Day 3, D7 = Day 7.

Subtle phenotype changes were characterized over seven days for PC3, DU145, and LNCaP cells cultured in 3D suspensions in Figs. 4, 5, and S4. The EIS was reported as an average impedance. Here the average impedance spectra are provided for each cell type at each measured time point. Large differences in Day 3 and Day 7 timepoints were observed for all cell lines.

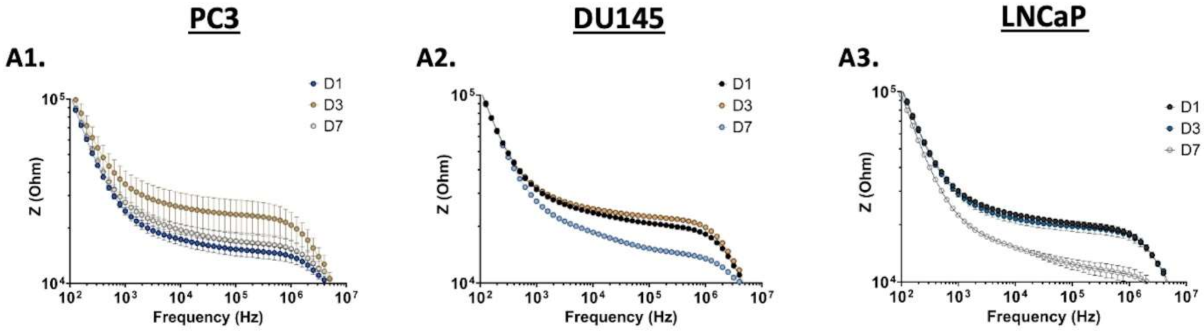

Figure S5. EIS spectra of (A1) PC3, (A2) DU145, and (A3) LNCaP cells cultured in 3D conditions overtime.

The chemoresistance of PC3 cells was characterized in Fig. 6. The EIS was reported as an average impedance. Here the average impedance spectra are provided. After the 48 hours, there was little change in impedance of the 2D monolayer and 3D suspension cells treated with 0 μM, 5 μM, and 10 μM of nigericin. After 96 hours, the magnitude of the impedance spectra increased, demonstrating a cellular response (i.e., cell death) to nigericin.

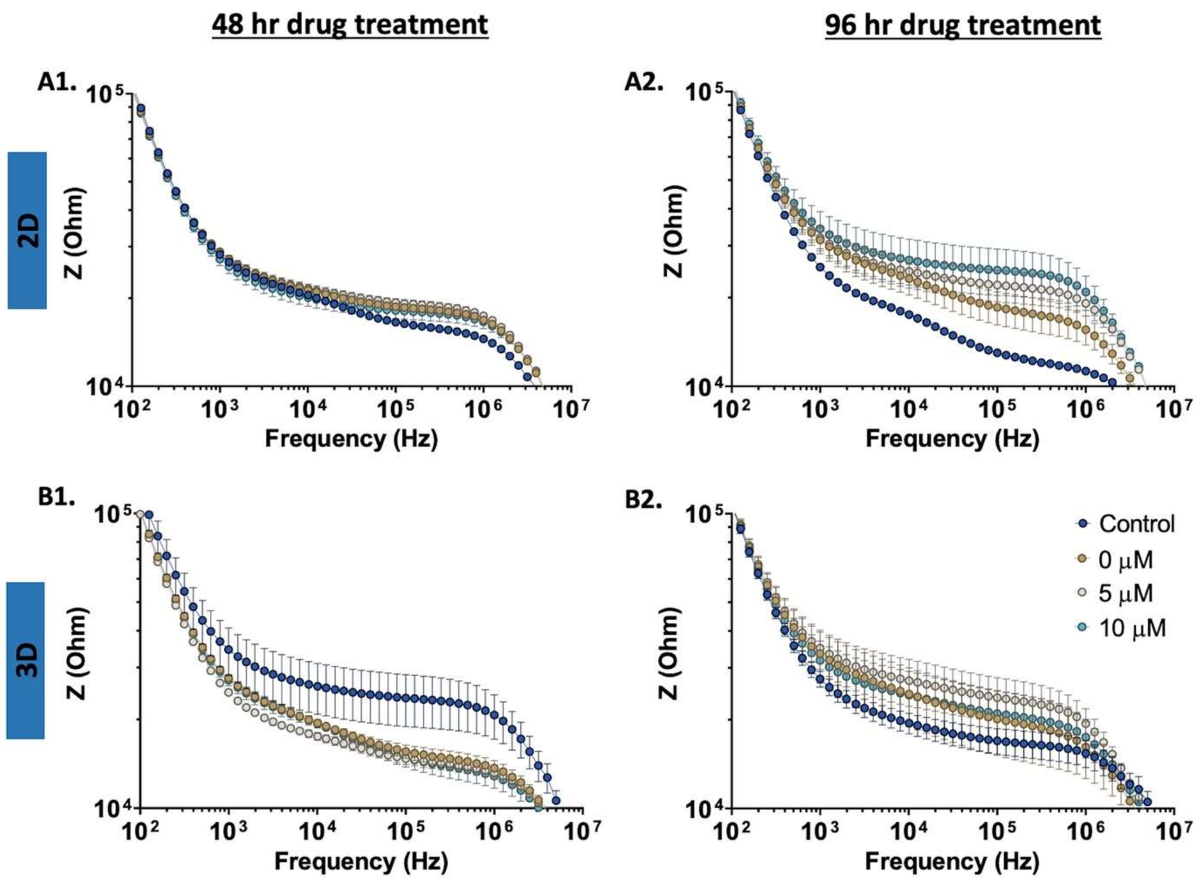

Figure S6. EIS spectra of PC3 cells treated with 0 μM, 5 μM, 10 μM of nigericin. (A1) 2D monolayer cells after the 48-hour drug treatment. (A2) 2D monolayer cells after the 96-hour drug treatment. (B1) 3D suspension cells after the 48-hour treatment. (B2) 3D suspension cells after the 96-hour treatment. 0 μM included only DMSO.
